# Multisensory integration approach, cognitive domains, meaningful learning: reflections for undergraduate nursing education

**DOI:** 10.1590/1980-220X-REEUSP-2021-0381

**Published:** 2022-04-11

**Authors:** Gabriela Feitosa Esplendori, Rika Miyahara Kobayashi, Vilanice Alves de Araújo Püschel

**Affiliations:** 1 Universidade de São Paulo, Escola de Enfermagem, São Paulo, SP, Brazil.; 2 Instituto Dante Pazzanese de Cardiologia, São Paulo, SP, Brazil.

**Keywords:** Education, Nursing, Learning, Teaching Materials, Cardiology, Students, Nursing, Perception, Educación en Enfermería, Aprendizaje, Materiales de Enseñanza, Cardiología, Estudiantes de Enfermería, Percepción, Educação em Enfermagem, Aprendizagem, Materiais de Ensino, Cardiologia, Estudantes de Enfermagem, Percepção

## Abstract

Teaching with a multisensory approach helps students link new information to prior knowledge and understand relationships between concepts. This study aimed to reflect on convergences between the Multisensory Integration Approach Model with the Learning Assimilation Theory and Meaningful Retention with Bloom’s Cognitive Process Domain, and to propose a taxonomic table of lesson planning for teaching Acute Coronary Syndrome, considering the confluence of these references. The three frameworks consider the importance of students’ prior knowledge, the process of abstraction and generalization of knowledge, and the relationship between working and long-term memory. By observing such convergences and the taxonomic table produced, it is observed that teaching topics of interest to nursing undergraduate students, adopting the Multisensory Integration Approach Model as a taxonomic table component (pre-organizing or recall activities to arouse different sensory perceptions aligned with instructional objectives and forms of assessment), in the light of the Learning Assimilation Theory and Meaningful Retention, has the potential to favor the reception and processing of instructional content.

## RESUMO

Ensinar com abordagem multissensorial auxilia o aluno a vincular informações novas ao conhecimento prévio e a entender relações entre conceitos. O objetivo deste estudo é refletir sobre convergências entre o Modelo de Abordagem de Integração Multissensorial com a Teoria da Assimilação da Aprendizagem e da Retenção Significativas com o Domínio dos Processos Cognitivos de Bloom, e propor uma tabela taxonômica de planejamento de aula para o ensino de Síndrome Coronariana Aguda, tendo em vista a confluência desses referenciais. Os três referenciais consideram a importância do conhecimento prévio do aluno, do processo de abstração e generalização do conhecimento e o relacionamento entre memória de trabalho e de longo prazo. Ao observar tais convergências e a tabela taxonômica produzida, observa-se que ensinar temas de interesse para graduandos de enfermagem, adotando o Modelo de Abordagem de Integração Multissensorial como um componente da tabela taxonômica (atividades pré-organizadoras ou de *recall* para suscitar diferentes percepções sensoriais alinhadas aos objetivos instrucionais e formas de avaliação), à luz da Teoria da Assimilação da Aprendizagem e da Retenção Significativas, tem potencial para favorecer a recepção e o processamento de conteúdos de instrução.

## RESUMEN

La enseñanza con un enfoque multisensorial ayuda al alumno a vincular la nueva información con el conocimiento previo y a comprender las relaciones entre los conceptos. El objetivo de este estudio es reflexionar sobre las convergencias entre el Modelo de Enfoque de Integración Multisensorial con la Teoría de la Asimilación y Retención del Aprendizaje Significativo con el Dominio de los Procesos Cognitivos de Bloom, y proponer una tabla taxonómica de planificación de lecciones para la enseñanza del Síndrome Coronario Agudo, en vista de la confluencia de estas referencias. Los tres marcos consideran la importancia del conocimiento previo del estudiante, el proceso de abstracción y generalización del conocimiento, y la relación entre la memoria de trabajo y la memoria a largo plazo. Al observar tales convergencias y la tabla taxonómica producida, se observa que la enseñanza de temas de interés para los estudiantes de enfermería, adoptando el Modelo de Enfoque de Integración Multisensorial como componente de la tabla taxonómica (actividades previas a la organización o recordación para suscitar diferentes percepciones sensoriales alineadas con objetivos instruccionales y formas de evaluación), a la luz de la Teoría de la Asimilación y Retención del Aprendizaje Significativo, tiene el potencial de favorecer la recepción y procesamiento del contenido instructivo.

## INTRODUCTION

Education involves two distinct and integrated processes: teaching and learning. Learning is the process of entering, processing and storing information in the cognitive system, as well as any persistent change in behavioral attributes, produced by the action of experience in the central nervous system^(1)^.

Specifically, multisensory learning is a process that consists of learning a new subject through the use of two or more senses, which may include visual, auditory, tactile or synesthetic, olfactory, and gustatory sensation^(2)^.

Teaching consists of creating favorable conditions for learning to occur^(3)^. In particular, teaching with a multisensory approach provides additional ways of receiving information into students’ cognitive system by stimulating hearing, vision, touch, speech, taste, movement and action, helping students to link new information to prior knowledge and to understand the relationships between concept^(4)^.

The definitions of multisensory learning and teaching are consistent with the fact that, along the sensory pathways, relatively simple information is transformed into complex forms, the basis of cognition^(5)^.

Receptors for each of the sensory systems provide neural representation of the external world so that information from the sense organs flows centrally to the brain regions involved in cognition^(5)^. Moreover, interactions occur between the visual, auditory, and somatosensory cortex with the prefrontal cortex^(6)^, which supports several higher cognitive processes, including perception, memory, and metacognition^(7)^. It is known that low-level sensory integration (visual-auditory) or high-level sensory integration (visual-auditory-tactile-olfactory-gustatory) occurs in the human brain, involving coordination, attention, autonomic function, emotions, cognitive functions of high level and memory^(8)^.

Teaching focused on sensory integration plays a vital role in improving working memory^(2)^, which is understood as “a temporary network that sustains current processing contents” and has subsidiary systems (capable of retaining information based on speech and information related to visual perception) an executive center capable of manipulating the results of perceptual processing with information stored in long-term memory^(9)^.

Multisensory education training can enhance learning and support successful, creative and sustainable career development in complex work environments^(10)^, as multisensory processes are critical to perception, cognition, learning, and behavior^(6)^.

In undergraduate nursing education, some studies have been carried out to explore undergraduate students’ attention and sensory perceptions, developing teaching focused on a multisensory approach in classes using traditional^(11–13)^ or active methodologies^(14)^. In these studies, diversified resources were used, such as peas, chocolate and candy packaging^(12)^, multicolored buttons, gloves, ear protectors^(12)^, balloons, drawing of human heart on the classroom floor, cardboard, ribbons and colored cards^(13)^, bright and colorful poster-size visual collages, children’s photographs and images of objects^(14)^.

The authors of the aforementioned studies describe that such resources and approaches contributed to student learning, by favoring the understanding of teaching topics and key concepts^(11–13)^, by facilitating information transfer, problem solving and critical thinking^(13)^, and helping to form thoughts and ideas^(14)^. In this perspective, research on multisensory learning is encouraged so that learning mechanisms and processes within natural settings can be better understood^(15)^. Despite providing additional ways for students to receive information, multisensory teaching is widely applied in the context of teaching and learning children with language and learning disorders, and little explored in depth in other subjects^(16)^.

A way to stimulate sensory perception and encourage students’ participation in the construction of their learning, in an expository class with dialogue inside a classroom, is proposed by Prasannakumar and Saminathan^(8)^, who describe the Multisensory Integration Approach Model (MIAM) as consisting of seven steps. These steps relate to “how” to conduct a class using the multisensory integration approach.

In step 1, “Relating new information”, there is a discussion in the room to identify previous knowledge; in step 2, “Focusing attention to the information”, there is use of innovative illustrations, gestures, provision of information through visual, auditory and tactile discrimination and repetition of ideas; in step 3, “Developing sensory connection”, there is use of verbal and non-verbal cues using visual and auditory sensation and then tactile sensation; in step 4, “Organizing the information”, graphic organizers are used through visual and auditory resources, with the objective of clarifying and clarifying students’ concepts; in step 5, “Expanding sensory images”, there is provision of role-play and simulation techniques, analogies and metaphors to improve students’ sensory image and provision of tasks that require hand-eye coordination to integrate concepts; in step 6, “Structuring the information”, a problem is presented to be solved using the auditory system and images, and activities are provided for tactile conceptualization and formulation of hypotheses and generalizations about concepts; in step 7, “Practicing recall”, working memory is considered with repetition of information, mnemonic techniques are used and students must be able to recall and recognize information using auditory and visual memory^(8)^.

Prasannakummar and Saminathan^(17)^ carried out an intervention study for science teaching, with an experimental group (using MIAM) and a control group (traditional teaching method), and concluded that MIAM improved student performance and contributed to a “meaningful and joyful learning”, suggesting that multisensory integration be used at all educational levels, in order to optimize learning.

MIAM has the potential to favor/optimize students’ learning. When inserted in lesson planning, in line with instructional objectives and the desired assessment format, it is possible to create an external environment in the classroom that arouse different sensory perceptions, with the potential to help in the existing relationship between working memory and long-term memory (new subjects and previous knowledge). Indeed, activities that require movement and the sense of touch make students dynamically participate in their education, rather than passively absorbing information through their eyes and ears^(18)^.

The observation of MIAM’s seven steps, in a reflective perspective, allows an approximation of it with David Ausubel’s Learning Assimilation Theory and Meaningful Retention (when observing the way of conducting the seven steps), and of MIAM with Bloom’s Taxonomy (when looking at the verbs of the seven steps).

Therefore, the present theoretical study aims to: 1) Reflect on the existing convergences between MIAM with David Ausubel’s Learning Assimilation Theory and Meaningful Retention (LATMR) and with Bloom’s Cognitive Process Domain, in order to expand the look at lesson planning (construction of instructional objectives, activities to be carried out during the class, learning assessment formats); 2) Propose a Taxonomic Table of lesson planning for teaching Acute Coronary Syndrome, in the confluence of these frameworks (MIAM, LATMR, and Bloom).

To this end, four sections will be presented below. The first will discuss on the LATMR; the second, on Bloom’s Cognitive Process Domain, to later discuss, in the third section, convergences between them and propose (in the fourth section) an educational activity with a Taxonomic table of lesson planning for teaching Acute Coronary Syndrome for nursing students, at the confluence of these frameworks.

## LEARNING ASSIMILATION THEORY AND MEANINGFUL RETENTION

The LATMR is defined by its author, David Ausubel, as a theory of “the way in which human beings apprehend and retain large sets of organized material in the classroom and similar learning environments”^(19)^.

David Ausubel points out that it is likely that cognitive factors and interpersonal motivation interact, influencing the learning process, and that this process involves relationships with other individuals. However, he cuts the scope of his theory into cognitive aspects, such as principles of cognitive organization and interaction and cognitive mechanisms^(19)^. Therefore, the meaningful learning described by David Ausubel consists in selective anchoring (linking) of the learning material to relevant ideas, existing in students’ cognitive structure, and in the interactions between them, in a non-arbitrary way, where the meaning of what was introduced emerges as a product of interaction as well as the connection of the new meaning with corresponding ideas in the memory interval (retention)^(19)^.

Anchored ideas are called subsumptions, and “factors that influence the clarity and stability of subsuming ideas are likely to include repetition (recall), use of copies, and multicontextual exposition”^(19)^.

Ausubel^(19)^ highlights that students’ prior knowledge is essential for discussion of a new subject, and that the repetition of a subject improves learning in two different ways. One is that repetition consolidates learned material more effectively when performed shortly after initial learning (before much of the forgetting has taken place). Furthermore, repetition can act in a scenario of ambiguous and unstable subsumptions, which provide weak anchoring for new information/concepts, constituting a factor influencing the clarity and stability of subsumption ideas^(18)^.

Another point of LATMR, which is worth mentioning in the scope of this study, is the observation that learners’/students’ cognitive structure can be systematically influenced by the methods of content presentation, when using an organized and pre-tested instructional material^(19)^.

There are two important principles in expository teaching: principles of progressive differentiation and integrative reconciliation in instructional materials so that the former recognizes that learning and its retention are hierarchical, i.e., it occurs by performing abstraction, then generalization and inclusion. Integrative reconciliation, on the other hand, is facilitated in expository teaching if teacher and/or teaching materials explicitly anticipate and counterattack the confusing similarities and differences between new ideas and ideas already anchored in learners’ cognitive structure^(19)^.

The use of pre-organizers at the beginning of a class/training can provide an anchor-shaped structure for students to grasp the new material^(20)^, playing the role of mediator between the particular content of the task of learning and the more general content of ideas potentially anchored in learners’ cognitive structure^(19)^. In this regard, pre-organizers, understood as a general concept or pedagogical mechanism, can be offered as a diagram, flowchart, a general word or a sentence^(20)^.

In addition to using pre-organizers, teachers can maximize learning and retention of their students’ learning in different ways, either by making regular stimulus changes and using color and movement, or by making changes in teaching methods, using gestures to help students to focus and identify important content for their learning^(20)^.

## BLOOM’S TAXONOMY: COGNITIVE PROCESS DOMAIN

Bloom’s Taxonomy of Cognitive Process Domain is used in Higher Education^(21,22)^. It is an instrument to develop learning objectives, to direct the teaching and learning process, and to assess whether or not the desired mental action has been demonstrated by students^(23)^, as it favors a hierarchical organization according to levels of complexity and desired and planned cognitive development goals^(24)^.

**Figure 1.**
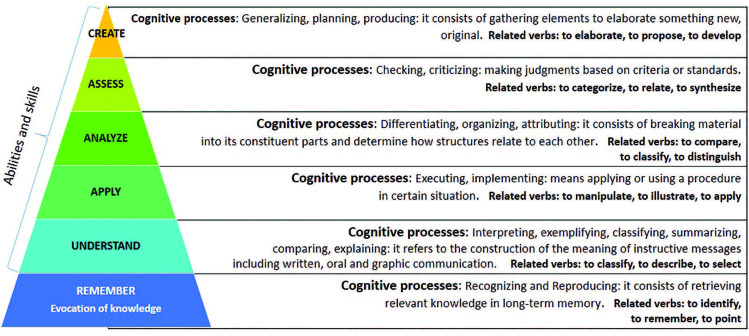
Bloom’s Cognitive Process Domain categories and verbs for learning assessment. São Paulo, 2021.

Source: Adapted from Anderson LW, et al.^(25)^.

For Bloom, everyone learns, but there are differences regarding the level of depth and abstraction of embedded knowledge^(24)^. Cognitive process domain categories involve the acquisition of new knowledge, as well as intellectual development, skills and attitudes, so that the six categories are presented in a hierarchical structure in complexity, from the simplest to the most complex, from the concrete to the abstract, where to ascend in a category it is necessary to obtain adequate performance in the previous category^(24)^, as shown in Figure 1.

The category “Remember” is closely related to the process of retaining the content presented in long-term memory, while the other five are related to the transfer process^(25)^.

An important aspect of the aforementioned Taxonomy refers to the degree of correspondence between instructional objectives, instructions (activities) and assessment, a degree that is verified by comparing objectives with assessment, objectives with instruction, and instruction with assessment. Such comparisons are easier to perform when the Taxonomic Table, so called by its authors, is used; however, its format is a table, in which the columns illustrate the cognitive processes and the lines the dimension of knowledge^(25)^.

The dimensions of knowledge, according to Bloom’s Taxonomy, include factual, conceptual and procedural knowledge. Factual knowledge means knowledge of discrete and isolated content elements, which includes knowledge of terminology and knowledge of specific details and elements^(25)^.

Procedural knowledge means “Knowledge of how to do something”, including knowledge of skills and algorithms, techniques and methods, as well as knowledge of the criteria used to determine and/or justify “when to do what” within domains and specific subjects. By conceptual knowledge, we mean the “more complex and organized” forms of knowledge, and includes knowledge of classifications and categories, principles and generalizations, theories, models and structure^(25)^.

## CONVERGENCES BETWEEN MIAM, LATMR AND BLOOM TAXONOMY’S COGNITIVE PROCESS DOMAIN

The elements that reflect approximations and convergences between MIAM and LATMR, and between MIAM and Bloom’s Cognitive Process Domain, consist of observation and comparison of MIAM steps with the concepts explained and defended in LATMR and with Bloom’s Cognitive Processes.

LATMR and MIAM emphasize the importance of students’ prior knowledge and the repetition of ideas (recall). To this end, copies and pre-organizers/graphic organizers can be used and information can be offered through organized and pre-tested instructional materials with visual, auditory and tactile discrimination, or through verbal and non-verbal cues that involve students to paraphrase information with sensations coming from the sense organs. One can also carry out the attack and counterattack of similarities and differences and techniques of dramatization, simulation, analogies, metaphors and hand-eye coordination, facilitating progressive differentiation through the presentation of a problem to be discussed using auditory system, images and tact to form hypotheses and generalize.

**Figure 2.**
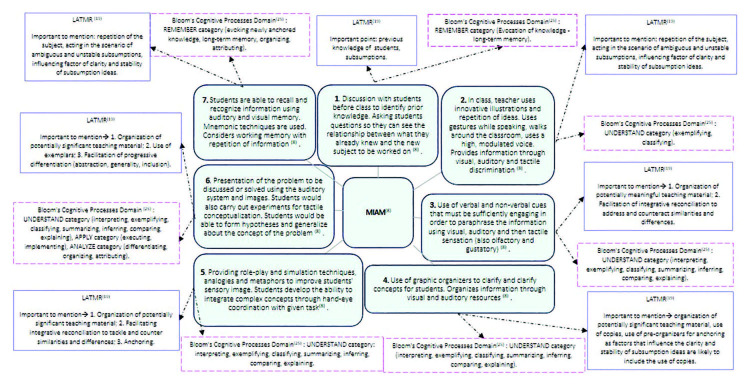
Convergences between the Multisensory Integration Approach Model, the Learning Assimilation Theory and Meaningful Retention and Bloom’s Cognitive Process Domain, São Paulo, 2021.

Source: Adapted from Prasannakumar S, Saminathan B^(8)^; Ausubel DP^(19)^; Anderson LW, et al.^(25)^.

Bloom’s Cognitive Process Domain categories and MIAM are similar, as MIAM steps are configured as a “way” of working the instructional content in the classroom (to favor content reception or processing, aiming at the reach of different cognitive abilities, according to the cognitive processes involved), or are configured as a necessary condition (importance of evoking previous knowledge) to reach the desired instructional objectives. Figure 2 shows in detail the steps of MIAM and its approximations to LATMR and Bloom’s Cognitive Process Domain.

The following is a proposal for the application of confluences illustrated in Figure 2 in the context of undergraduate students’ teaching and learning.

## PROPOSAL FOR TEACHING ACUTE CORONARY SYNDROME FOR NURSING UNDERGRADUATE STUDENTS WITH ACTIVITIES AIMED AT SENSORY INTEGRATION

For the purposes of reflection and approximation of MIAM to the educational scenario in nursing, in the light of LATMR and Bloom’s Cognitive Process Domain, a topic of global relevance for teaching nursing students was listed, Acute Coronary Syndrome (ACS), as ischemic heart disease is the leading cause of death in the world, accounting for 8.9 million (16%) deaths in 2019^(26)^.

It is noteworthy that this proposal aims to make connections between instructional objectives, activities with multisensory integration approach performed in the classroom and learning assessment, considering as a target audience nursing undergraduates who have already attended subjects of anatomy, physiology and human histology and pharmacology. The intention is not to replace strategies and resources that each teacher can adopt in their lesson planning, but to aggregate and raise considerations about another way to teach this theme, according to its insertion in the subject focused on nursing care for adults and older adults.

In more detail, to design the suggested teaching proposal, the following steps were followed: 1) Definition of the strategy to be adopted to conduct the proposal (dialogued exposition); 2) Adoption of LATMR for the development of the proposal, by understanding nursing students as actors in their learning process, with previous knowledge about anatomy, physiology, human histology and pharmacology; 3) Use of Bloom’s Taxonomy for planning the proposal, as it is a didactic-pedagogical instrument that helps in the elaboration of instructional objectives and in the observation of the correspondence between them with the instruction carried out and assessment learning items^(25)^; 4) Determination that MIAM will be used specifically as a guide for the development of instructional activities aimed at sensory integration to practice recall or to offer pre-organizers (elements described in LATMR as factors that favor the learning process).

The principle adopted for the teaching proposal consisted of conducting the class with a dialogued expository strategy in the on-site modality, using instructional resources that require low financial investment (in order not to compromise its implementation and replication). It is important to highlight that the suggested activities can be improved (being only an initial draft) and developed in a dialogued exhibition class, with the use of multimedia resources and household/stationery materials. These materials may be provided by students or adapted/replaced by similar objects from teachers’ prior request.

**Chart 1.**
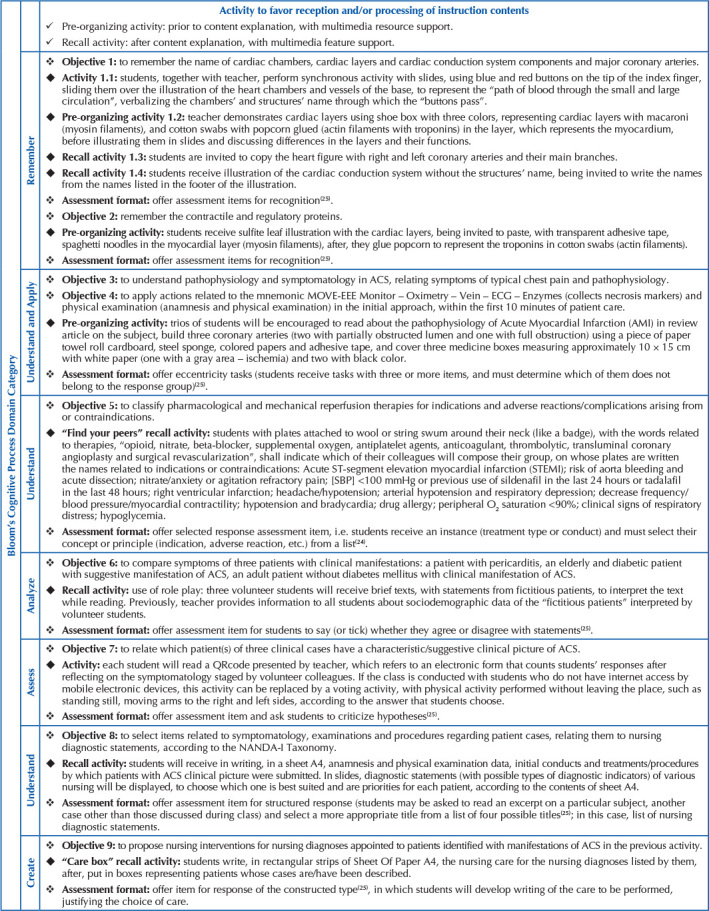
Proposal of instructional objectives, corresponding to activities with multisensory integration approach and assessment formats. São Paulo, Brazil, 2021.

Source: Prepared by the authors.

Chart 1 describes the instructional objectives, their respective activities with a multisensory integration approach and ways of assessing learning in the light of LATMR, considering Bloom’s Taxonomy of Cognitive Processes Dimension.

**Chart 2.**
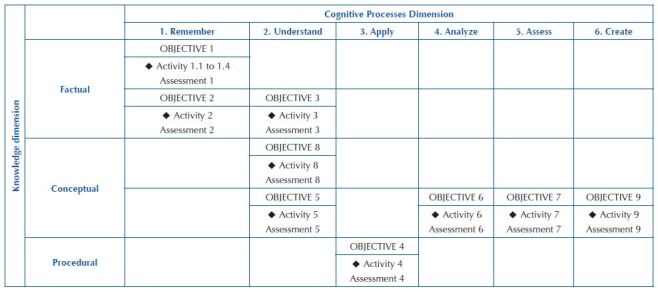
Proposal for a taxonomic table of class on Acute Coronary Syndrome for nursing undergraduate students. São Paulo, Brazil, 2021.

Source: Prepared by the authors. Caption: ◆ Use of the Multisensory Integration Approach Model, in order to favor reception and/or processing of instructional contents.

After this stage of elaboration of instructional objectives for activities with multisensory integration approach and assessment formats, a taxonomic table was built, which summarizes the framework/correspondence of instructional objectives, planned activities and assessment formats in the dimensions of knowledge and dimensions of cognitive processes, simultaneously, according to Chart 2.

Looking the taxonomic table (Chart 2) offers teachers an overview of the teaching process, enabling them to visualize the type of assessment that is most coherent with the instructional objective to be achieved and which activity to develop to achieve instructional objectives distributed among knowledge and cognitive process domains.

## CONCLUSION

Convergences between MIAM, LATMR and Bloom’s Taxonomy Cognitive Process Domain consist of the fact that these three frameworks consider the importance of students’ prior knowledge, the process of abstraction and depth of knowledge, and the relationship between working and long-term memory.

Such convergences can be perceived in a taxonomic table for teaching ACS, in which MIAM is configured as a component of this table, aiming at sensory perceptions during the conduct of a class with the performance of pre-organizing or recall activities, which provide different ways to promote the reception and processing of learning contents within classroom.

In the context of undergraduate nursing, randomized clinical trials are necessary to verify the real effect of teaching, using MIAM in undergraduate students’ learning and based on guiding frameworks, such as Bloom’s Taxonomy in light of LATMR. Studies with qualitative methodology are also indispensable to understand teaching with this approach, from undergraduate students’ perspective, regarding the affective aspects and interpersonal motivation that permeate the learning process.

## ASSOCIATE EDITOR

Paulino Artur Ferreira de Sousa
